# Psychological Distress Among Oral and Maxillofacial Surgery Professionals: A Systematic Review

**DOI:** 10.7759/cureus.108390

**Published:** 2026-05-06

**Authors:** Kavitha Swaminathan, Subbalekshmi T, Tanu Nangia, Ganesh Rajendran, Tabassum Tayab, Selvakumar Haridoss

**Affiliations:** 1 Pediatric and Preventive Dentistry, Sri Ramachandra Institute of Higher Education and Research, Chennai, IND; 2 Pediatric Dentistry, Malabar Dental College and Research Centre, Edappal, IND; 3 Pediatric Dentistry, Manav Rachna Dental College, Faridabad, IND; 4 Pediatric Dentistry and Preventive Dentistry, Al-Baha University, Al-Baha, SAU; 5 Al Hilal Hospitals, Specialist Pediatric Dentist, Muharraq, BHR; 6 Pedodontics and Preventive Dentistry, Sri Ramachandra Institute of Higher Education and Research, Chennai, IND

**Keywords:** anxiety, good health and well-being, occupational burnout, oral and maxillofacial surgery, perceived stress, psychological distress, residency training, systematic review

## Abstract

Psychological distress among oral and maxillofacial surgery (OMFS) professionals is increasingly recognized as a concern due to the demanding nature of training and clinical practice. This systematic review aimed to synthesize available evidence on perceived stress and psychological distress among OMFS surgeons, residents, and trainees. A comprehensive search of PubMed/MEDLINE, Scopus, Web of Science, and Google Scholar was conducted up to April 10, 2026. Six cross-sectional studies comprising 1,149 OMFS participants met the inclusion criteria. Due to substantial heterogeneity in measurement instruments, study populations, and outcome reporting, a meta-analysis was not performed, and findings were synthesized narratively in accordance with Synthesis Without Meta-analysis (SWiM) principles. Across studies, a consistently high burden of psychological distress was observed, with moderate-to-severe anxiety reported in a substantial proportion of trainees and nearly half of surgeons classified as at risk of burnout. Residents demonstrated higher perceived stress levels compared to practicing surgeons. Workload-related factors, including long working hours and frequent on-call duties, were strongly associated with increased stress and burnout risk. In contrast, psychosocial factors such as academic pressure and workplace dynamics further contributed. Despite variability in measurement approaches, a consistent relationship was observed between increased occupational demands and reduced well-being. The findings highlight the need for standardized assessment tools and targeted interventions addressing both individual and institutional factors to improve mental health outcomes and support workforce sustainability in OMFS.

## Introduction and background

Occupational stress is a well-recognized determinant of health and professional well-being among healthcare workers, arising from sustained high job demands, limited autonomy, and insufficient organizational support [1]. When such stress is chronic and unresolved, it may lead to burnout, a psychological syndrome characterized by emotional exhaustion, depersonalization, and reduced personal accomplishment [2]. Burnout has been associated with reduced job satisfaction, impaired clinical performance, and, in surgical specialties, an increased risk of medical errors [3]. Oral and maxillofacial surgery (OMFS) presents a combination of factors that heighten this risk, including prolonged training requirements, involvement in complex procedures such as trauma and oncology, and frequent exposure to patients in acute physical and emotional distress [4,5]. These characteristics make OMFS professionals particularly vulnerable to cumulative stress across all career stages.

Evidence of psychological distress among OMFS professionals has increased over the past decade. A systematic review of dental students found higher perceived stress among those in clinical training than in non-clinical pathways, with academic pressure and workload identified as key contributors [6]. Within OMFS, studies of trainees have shown that more than 60% experience burnout during training, with factors such as gender, workload, and financial stress contributing to this burden [5]. Among practicing surgeons, approximately 40% have been reported to meet burnout criteria, with burnout strongly associated with career dissatisfaction [7]. Despite this growing body of evidence, findings remain fragmented across different career stages, geographical settings, and measurement tools, including the Perceived Stress Scale (PSS-10), Hospital Anxiety and Depression Scale-Anxiety subscale (HADS-A), Maslach Burnout Inventory (MBI), and Physician Well-Being Index (WBI), limiting comparability and a clear understanding of the overall burden.

Currently, no systematic review has comprehensively synthesized evidence on perceived stress and psychological distress across the full spectrum of OMFS career stages. Given the implications for workforce sustainability, training quality, and patient safety [3,8], a structured synthesis is needed. Therefore, this systematic review aimed to identify, critically appraise, and narratively synthesize studies reporting validated measures of perceived stress or psychological distress among OMFS professionals, and to examine factors associated with these outcomes across career stages and practice settings.

## Review

Methods

Study Design and Protocol Registration

This systematic review was conducted in accordance with the Preferred Reporting Items for Systematic Reviews and Meta-Analyses (PRISMA 2020) guidelines [9]. The review protocol was prospectively registered in the International Prospective Register of Systematic Reviews (PROSPERO) prior to data collection under the registration number CRD420261365168. The completed PRISMA checklist is provided as a supplementary file.

Eligibility Criteria

Studies were included or excluded based on the following prespecified criteria, as summarized in Table 1.

**Table 1:** Pre-specified eligibility criteria for study inclusion and exclusion OMFS: oral and maxillofacial surgery, OMS: oral and maxillofacial surgery, PSS-10: Perceived Stress Scale, HADS-A: Hospital Anxiety and Depression Scale-Anxiety subscale, MBI: Maslach Burnout Inventory, WBI: Physician Well-Being Index, GDES30: Graduate Dental Environment Stress Scale

Domain	Inclusion criteria	Exclusion criteria
Population	OMFS/OMS surgeons, residents, postgraduate trainees, and fellows at any career stage. In mixed-specialty studies, OMFS-specific data must be reported and extracted separately.	Non-OMFS dental or medical professionals where OMFS data were not separately extractable.
Study design	Cross-sectional surveys, cohort studies, case-control studies, and pilot analytical surveys that reported quantified outcome data were included.	Systematic reviews, narrative reviews, editorials, case reports, and conference abstracts without full data.
Outcome	Perceived stress or psychological distress was assessed using a validated or structured instrument (e.g., PSS-10, HADS-A, MBI, WBI, GDES30). Burnout co-reported with a stress or distress measure was eligible.	Studies reporting only career or job satisfaction without validated instruments for stress or distress. Studies reporting burnout alone with no co-reported stress or distress measures.
Language	English only.	All other languages.

Information Sources and Search Strategy

A systematic search was conducted across four electronic bibliographic databases: PubMed/MEDLINE, Scopus, Web of Science (Core Collection), and Google Scholar (first 100 hits). The search spanned from database inception to April 10, 2026. No date restrictions or language filters were applied during the database search; however, the eligibility criteria excluded English-language publications at the screening stage. The search strategy included combinations of keywords and Boolean operators, such as (“oral and maxillofacial surgery” OR “OMS” OR “OMFS”) AND (“stress” OR “psychological distress” OR “burnout” OR “anxiety”), to ensure comprehensive retrieval of relevant studies.

The search strategy was structured around three conceptual domains combined using Boolean operators: population (oral and maxillofacial surgery, OMS, OMFS, maxillofacial surgeon, oral surgery resident), outcome (stress, perceived stress, psychological distress, anxiety, burnout, emotional exhaustion, well-being, mental health), and context (occupational, workplace, residency, training, postgraduate, surgeon, practitioner). The full electronic search strategy for each database is provided as a supplementary table.

To supplement the database searches, the reference lists of all the included studies were manually screened for additional relevant publications. Forward citation tracking was performed for the key studies included using Google Scholar.

Study Selection

All records retrieved from the four databases were imported into Rayyan (rayyan.ai, Qatar Computing Research Institute, Doha, Qatar), a web-based collaborative systematic review management platform [10]. Automatic duplicate detection was applied within Rayyan and supplemented by a manual verification.

Screening was conducted independently by two reviewers in two sequential stages: title and abstract screening followed by full-text eligibility assessment.

Disagreements at either screening stage were resolved through discussions between the two reviewers. In cases where consensus could not be reached through discussion, a third reviewer was consulted for adjudication. The third reviewer's final decision was binding on the two other reviewers.

Data Extraction

Data were independently extracted from the included studies by two reviewers using a standardized extraction form developed by the review team prior to data collection. Discrepancies between reviewers were resolved through discussion and cross-checking with the source publication. The following data were extracted from each included study: study characteristics (first author, year of publication, country, journal, and study design); population details (professional category, sample size, gender distribution, and training level or career stage); setting (practice context and data collection period, including whether conducted during or after the COVID-19 pandemic); outcome instruments (name of the validated or structured tool, subscales used, scoring method, and reported psychometric properties); key findings (mean scores with standard deviations, prevalence estimates, burnout risk classifications, or other reported stress or distress metrics); and associated factors (variables significantly associated with stress or psychological distress, particularly from multivariate analyses). When outcome data were not reported in sufficient detail, the corresponding authors were not contacted due to time and resource constraints, a limitation acknowledged in the review.

Risk of Bias Assessment

The methodological quality of each included study was assessed independently by two reviewers using the Joanna Briggs Institute (JBI) Critical Appraisal Checklist for Analytical Cross-Sectional Studies (8-item version) [11]. This tool was selected because all six studies used a cross-sectional survey design. Each criterion was judged independently by two reviewers as follows: yes (criterion met), no (criterion not met), unclear (insufficient information to judge), or not applicable. The overall risk of bias for each study was classified as low, low-to-moderate, or moderate, with particular weight assigned to criteria addressing measurement validity (Q3, Q4, Q7) and confounder control (Q5, Q6), as these were the domains most likely to vary across the included studies. Disagreements between the reviewers were resolved through discussion. Where consensus could not be reached, the same third reviewer consulted during the study selection provided adjudication.

Data Synthesis

A quantitative meta-analysis was initially planned per the PROSPERO-registered protocol; however, it was not performed due to substantial heterogeneity among the included studies. Heterogeneity was observed across measurement, population, and contextual domains.

Measurement heterogeneity: The six included studies used five distinct instruments: PSS-10 [12], HADS-A [13], WBI [14], GDES30 combined with the Career Satisfaction Scale [15-17], and non-validated self-report 0-100 scales, each measuring related but non-equivalent constructs. Outcome metrics included means on continuous scales, binary burnout risk classifications, Likert index scores, and odds ratios from the logistic regression. These differences precluded the transformation into a common effect size.

Population heterogeneity: The included studies recruited distinct OMFS populations: residents only (three studies), a mixed cohort of surgeons and residents (one study), and academic surgeons only (one study). These groups differ substantially in occupational exposure, seniority, and training demands, rendering pooled estimates clinically uninterpretable.

Contextual heterogeneity: Data were collected during the pre-pandemic (2017-2019), early pandemic (April-May 2020), and post-pandemic (2021-2024) periods. The COVID-19 pandemic operated as a major contextual confounder that varied systematically across studies and could not be statistically adjusted for in a pooled analysis.

These differences reflect conceptual and statistical non-equivalence across studies, thereby precluding meaningful quantitative pooling. Given this heterogeneity, a structured narrative synthesis was conducted in accordance with the Synthesis Without Meta-analysis (SWiM) reporting framework [18]. Studies were grouped by primary outcome domain (perceived stress, anxiety, and burnout risk) and summarized descriptively. Cross-study comparisons were made where instrument comparability was permitted, with explicit acknowledgment of construct differences where they were not.

Results

Study Selection

The systematic database search conducted on April 10, 2026, identified 493 records across four databases (PubMed/MEDLINE: 143; Scopus: 206; Web of Science: 44; Google Scholar: 100), with one additional record identified through citation tracking, yielding a total of 494 records. Following import into Rayyan and removal of duplicates (n = 162), 332 unique records underwent title and abstract screening. Of these, 305 records were excluded because they did not meet the predefined eligibility criteria.

**Figure 1:**
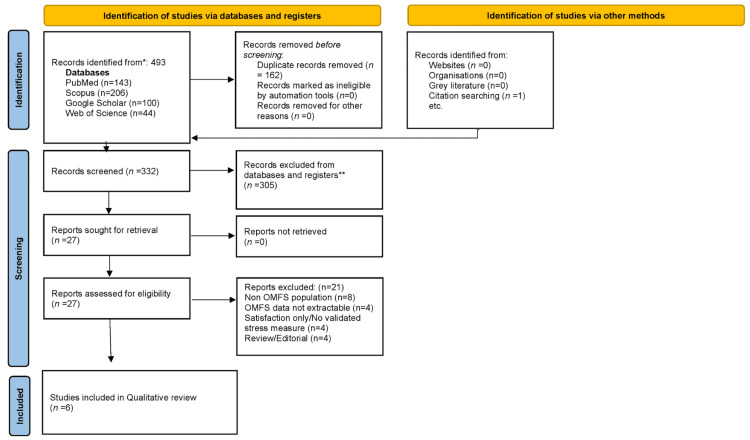
PRISMA flow diagram of the study selection process PRISMA: Preferred Reporting Items for Systematic Reviews and Meta-Analyses

A total of 27 full-text articles were assessed for eligibility. Of these, 21 studies were excluded for the following reasons: non-OMFS population (n = 8), OMFS data not separately extractable (n = 5), outcomes limited to satisfaction without validated stress/distress measures (n = 4), and review/editorial articles (n = 4). Finally, six studies met the eligibility criteria and were included in the qualitative synthesis. The PRISMA 2020 flow diagram is shown in Figure 1.

Characteristics of Included Studies

The six included studies collectively comprised 1,149 OMFS-specific participants and were conducted between 2018 and 2024 across the United States (n = 5) and Saudi Arabia (n = 1). All studies employed a cross-sectional survey design, and no cohort or case-control studies met the eligibility criteria.

The study populations varied across the included studies. Three studies included only OMFS residents, one included both surgeons and residents, one focused on academic OMFS surgeons, and one included OMFS residents as a subgroup within a multispecialty cohort.

The sample sizes ranged from 108 to 300 participants, with response rates varying widely from 20% to 95.6%. The response rate for the OMFS subgroup in one study was not calculable owing to indirect recruitment methods.

A range of validated and non-validated instruments were used to assess outcomes, including validated tools (HADS-A, PSS-10, WBI), composite or derived indices (GDES30), and non-standardized self-report scales. A detailed summary of the study characteristics is provided in Table 2.

**Table 2:** Characteristics of the included studies HADS-A: Hospital Anxiety and Depression Scale-Anxiety subscale, MBI: Maslach Burnout Inventory, PSS-10: Perceived Stress Scale, WBI: Physician Well-Being Index, GDES30: Graduate Dental Environment Stress Scale, AAOMS: American Association of Oral and Maxillofacial Surgeons, AACMS: American Academy of Craniomaxillofacial Surgeons, CODA: Commission on Dental Accreditation

Study (year)	Country	Study design	Population	Sample size (N)	Instrument(s) used	Outcome type	Response rate
Alkindi et al. (2020) [19]	Saudi Arabia	Cross-sectional survey	OMFS and OMFS residents	172	PSS-10; stress source questionnaire	Perceived stress (continuous + categorical)	95.6%
Al Atassi et al. (2018) [20]	USA	Cross-sectional survey	OMFS residents (AAOMS-accredited programs)	238	HADS-A; MBI (personal accomplishment)	Anxiety + burnout component	20%
Amin et al. (2021) [21]	USA	Cross-sectional survey	OMFS residents (CODA-accredited programs)	275	HADS-A; COVID-19-related items	Anxiety	24.3%
Milder et al. (2021) [22]	USA	Cross-sectional survey	Academic OMFS surgeons (AACMS fellows)	108	WBI	Burnout risk (binary classification)	61.1%
Smith et al. (2019) [23]	USA	Cross-sectional survey	OMFS residents (all accredited US programs)	300	Self-reported 0-100 scales	Stress, coping, satisfaction (non-validated)	25.4%
Inglehart et al. (2024) [24]	USA	Cross-sectional survey	Dental residents (OMFS subgroup n = 56/212)	212 (56 OMFS)	GDES30; Career Satisfaction Scale; Job Satisfaction Scale	Multi-domain stress + satisfaction	OMFS subgroup: not calculable

Perceived Stress and Psychological Distress Outcomes

Due to substantial heterogeneity in the measurement instruments and outcome reporting, the findings are presented by instrument type.

Anxiety (HADS-A): Two studies assessed anxiety using HADS-A [20,21]. In one study of 238 OMFS residents, 58.4% reported moderate-to-severe anxiety, with a mean score of 9.4 (SD 4.8). Severe anxiety was significantly more prevalent among female residents, and higher anxiety levels were strongly associated with reduced personal achievement [20]. Another study of 275 residents conducted during the COVID-19 pandemic reported that 41.8% experienced moderate-to-severe anxiety, with all participants reporting anxiety of some degree. Female sex, senior training level, and pandemic-related concerns (e.g., PPE adequacy and graduation uncertainty) were significantly associated with increased anxiety [21].

Perceived stress (PSS-10): Alkindi et al. assessed perceived stress using PSS-10 among 172 participants. Residents demonstrated significantly higher stress scores than surgeons (mean 20.61 vs. 17.51; P = 0.005). Most participants in both groups reported moderate stress levels, with a higher proportion of residents reporting moderate stress. Increased working days and longer on-call periods were significantly associated with higher stress scores [19].

Self-reported stress and satisfaction: Smith et al. used non-validated self-report scales to assess stress, coping, and satisfaction. Of the residents, 57% reported satisfaction, and 25% were dissatisfied. Increased working hours were associated with reduced job satisfaction. Access to mental health support significantly improved satisfaction, whereas perceived stigma surrounding mental health disclosure reduced satisfaction. Declines in health status were more pronounced among dissatisfied residents [23].

Burnout risk (WBI): One study assessed burnout risk among 108 academic OMFS surgeons using WBI. The mean score (2.21) was below the burnout threshold; however, 45.4% of the participants were classified as at risk. A high proportion of participants reported emotional exhaustion indicators, including feeling overwhelmed and emotionally hardened. Significant predictors of burnout risk included working ≥55 hours per week, undertaking more than 10 on-call shifts per month, having teaching as a primary professional responsibility, and being aged >40 years. Additionally, female surgeons demonstrated higher burnout risk scores [22].

Multi-domain stress (GDES30): Inglehart et al. evaluated multi-dimensional stress using GDES30-derived indices and found that OMFS residents reported significantly higher stress levels across multiple domains than residents of other dental specialties, particularly in personal life-related, faculty-related, and academic stress. Despite these elevated stress levels, OMFS residents reported high job satisfaction, with over 90% indicating that they would choose this specialty again. Additionally, exposure to discrimination and harassment was associated with increased stress and reduced satisfaction [24].

Synthesis of Findings (SWiM Approach)

A structured narrative synthesis was conducted in accordance with SWiM principles. Across all included studies, several consistent patterns emerged, including the high prevalence of stress and anxiety among OMFS trainees. Workload-related factors, such as long working hours and frequent on-call duties, were strongly associated with increased stress and burnout risk. Psychosocial and environmental factors, including academic pressure and workplace dynamics, also significantly contributed to psychological distress. Protective factors included access to mental health support and effective coping strategies. A consistent directional relationship was observed, wherein increased stress was associated with reduced satisfaction and well-being, increased workload was linked to higher psychological distress, and elevated stress and anxiety were associated with a greater burnout risk. These findings were consistent despite variations in measurement instruments.

Risk of Bias Assessment

**Figure 2:**
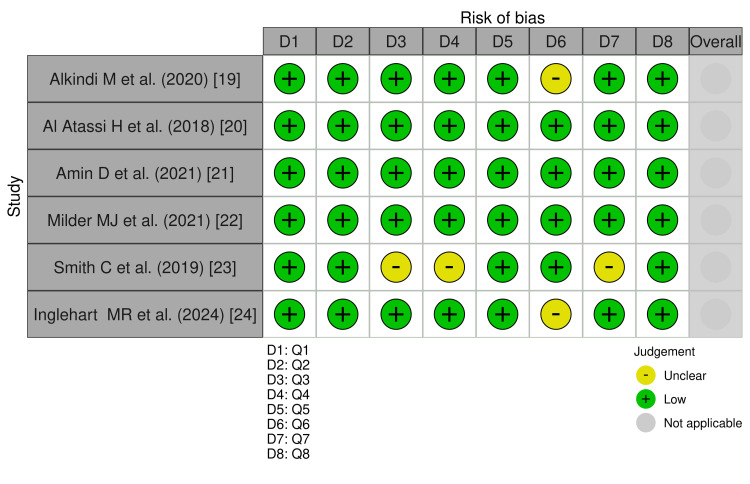
Item-level JBI traffic-light summary for the included cross-sectional studies JBI: Joanna Briggs Institute

The risk of bias was assessed using the JBI checklist. Of the included studies, three were rated as having a low risk of bias, two as having a low-to-moderate risk, and one as having a moderate risk. Most studies employed validated instruments and appropriate statistical analyses; however, limitations included the use of non-validated measures in one study, variable response rates, and the potential for non-response bias. A detailed assessment is shown in Figure 2.

Discussion

This systematic review synthesizes the evidence on perceived stress and psychological distress among OMFS professionals. A narrative synthesis approach was adopted due to substantial heterogeneity in measurement tools, study populations, and contextual factors. The included studies used non-equivalent instruments, such as PSS-10 [12], HADS [13], and WBI [14], as well as non-standardized measures that assessed related but distinct constructs. Additionally, variability across populations and differences in data-collection timing, particularly across pandemic periods, precluded a meaningful meta-analysis. These instruments measure related but non-equivalent constructs, which limits direct comparability across studies and precludes transformation into a common effect size.

The findings are consistent with the existing literature, which demonstrates high psychological distress in dental and surgical training environments. Dental trainees experience elevated stress due to academic demands and clinical workload [6,15], while burnout is widely reported among surgical trainees [6]. Within OMFS, both workload and training environment contribute to distress, with evidence suggesting that negative educational practices and workplace culture significantly influence burnout, independent of workload [25]. However, findings regarding demographic determinants such as gender remain inconsistent, with some studies reporting higher distress among female practitioners, while others show no significant differences [26,27].

Across the included studies, a consistently high burden of stress, anxiety, and burnout was observed [19-24]. Moderate-to-severe anxiety affected a substantial proportion of trainees, and a considerable proportion of surgeons were identified as being at risk of burnout. Residents reported higher stress levels than surgeons, suggesting that early stages of training are particularly vulnerable. Workload-related factors, such as long working hours and frequent on-call duties, were strongly associated with increased distress. In contrast, psychosocial factors, including academic pressure and workplace dynamics, further contributed to it. These findings align with established models that identify workload and organizational factors as key determinants of burnout [28,29].

This review has several strengths, including protocol registration, adherence to the PRISMA guidelines [9], dual-reviewer processes, and use of a standardized risk-of-bias tool [11]. However, the limitations of this study must be acknowledged. All the included studies were cross-sectional, which limited causal inference. Additionally, the predominance of studies from the United States may limit the generalizability of the findings to other healthcare systems with different training structures and sociocultural contexts. Heterogeneity in measurement tools restricted comparability, and reliance on self-reported data introduced potential response bias. Furthermore, restriction to English-language publications and the limited number of included studies may introduce publication bias and selective reporting. Variable response rates and the influence of the COVID-19 pandemic as a contextual factor further limit the generalizability of the findings [30].

Future research should prioritize longitudinal and multicenter studies to better understand the trajectory of psychological distress among OMFS professionals, particularly trainees. Standardization of outcome measures using validated instruments such as the PSS-10 and MBI would improve comparability and enable future meta-analyses. Additionally, evaluation of interventions targeting both individual and systemic factors, including workload regulation and improvement of training environments, is warranted.

## Conclusions

Psychological distress is highly prevalent among OMFS professionals, particularly among trainees. Both workload-related and psychosocial factors contribute significantly to stress, anxiety, and burnout among healthcare workers. Addressing these issues requires structured interventions at both the individual and institutional levels to improve clinician well-being and support sustainable clinical practice. The implications extend beyond clinician health, as burnout is associated with reduced quality of care and an increased risk of medical errors.

## Appendices

**Table 3:** Detailed search strategy for each database

Database	Search strategy	Date of search	Limits applied
PubMed/MEDLINE	("oral and maxillofacial surgery" OR OMFS OR OMS OR "maxillofacial surgeon" OR "oral surgery resident") AND ("stress" OR "perceived stress" OR "psychological distress" OR anxiety OR burnout OR "emotional exhaustion" OR "mental health") AND (occupational OR workplace OR residency OR training OR postgraduate OR surgeon OR practitioner)	April 10, 2026	No date restriction; English language applied during screening
Scopus	TITLE-ABS-KEY ("oral and maxillofacial surgery" OR OMFS OR OMS OR "maxillofacial surgeon" OR "oral surgery resident") AND TITLE-ABS-KEY ("stress" OR "perceived stress" OR "psychological distress" OR anxiety OR burnout OR "emotional exhaustion" OR "mental health") AND TITLE-ABS-KEY (occupational OR workplace OR residency OR training OR postgraduate OR surgeon OR practitioner)	April 10, 2026	No date restriction; English language applied during screening
Web of Science (Core Collection)	TS=("oral and maxillofacial surgery" OR OMFS OR OMS OR "maxillofacial surgeon" OR "oral surgery resident") AND TS=("stress" OR "perceived stress" OR "psychological distress" OR anxiety OR burnout OR "emotional exhaustion" OR "mental health") AND TS=(occupational OR workplace OR residency OR training OR postgraduate OR surgeon OR practitioner)	April 10, 2026	No date restriction; English language applied during screening
Google Scholar	("oral and maxillofacial surgery" OR OMFS OR OMS) AND ("stress" OR "psychological distress" OR anxiety OR burnout)	April 10, 2026	First 100 results screened; English language applied during screening

**Table 4:** PRISMA 2020 checklist PRISMA: Preferred Reporting Items for Systematic Reviews and Meta-Analyses

Section/topic	Item no.	Checklist item	Reported on page no.
Title	1	Identify the report as a systematic review	Page 1
Abstract	2	Provide a structured summary including background, objectives, methods, results, and conclusions	Page 1
Introduction	3	Describe the rationale for the review in the context of existing knowledge	Pages 1-2
	4	Provide an explicit statement of the objective(s) or question(s) the review addresses	Page 2
Methods	5	Specify inclusion and exclusion criteria for the review	Page 2 (Table 1)
	6	Specify all information sources (databases, registers, etc.) and date last searched	Page 2
	7	Present full search strategy including filters and limits	Page 2
	8	Describe methods used to select studies (screening process)	Page 3
	9	Describe data collection process	Page 3
	10a	List and define all outcomes for which data were sought	Page 3
	10b	List and define other variables (e.g., participant characteristics)	Page 3
	11	Specify methods used to assess risk of bias	Page 3
	12	Specify effect measures (if applicable)	Not applicable (no meta-analysis)
	13a	Describe synthesis methods	Pages 3-4
	13b	Methods used to prepare data for synthesis	Page 3
	13c	Methods used to tabulate or visually display results	Pages 4-5
	13d	Methods used to explore heterogeneity	Page 3-4
	13e	Sensitivity analyses	Not applicable
	14	Describe methods used to assess risk of bias due to missing results	Not reported
	15	Describe methods used to assess certainty of evidence	Not reported
Results	16a	Describe results of search and selection process	Page 4
	16b	Cite excluded studies and reasons for exclusion	Page 4
	17	Present characteristics of included studies	Pages 5-6 (Table 2)
	18	Present risk of bias in included studies	Page 7 (Figure 2)
	19	Present results of individual studies	Pages 6–7
	20a	Summarize results of syntheses	Page 7
	20b	Present results of statistical syntheses	Not applicable
	20c	Present investigations of heterogeneity	Page 3-4
	20d	Present sensitivity analyses	Not applicable
	21	Present reporting bias assessment	Not reported
	22	Present certainty of evidence	Not reported
Discusion	23a	Provide general interpretation of results	Page 8
	23b	Discuss limitations of included evidence	Page 8
	23c	Discuss limitations of review processes	Page 8
	23d	Discuss implications for practice and research	Page 8
Other information	24a	Provide registration information	Page 2 (PROSPERO)
	24b	Indicate where protocol can be accessed	Page 2
	24c	Describe amendments to protocol	Not reported
	25	Describe sources of funding	Page 9
	26	Declare competing interests	Page 9
	27	Describe availability of data, code, and materials	Not reported
